# Basal body structure and composition in the apicomplexans *Toxoplasma* and *Plasmodium*

**DOI:** 10.1186/s13630-016-0025-5

**Published:** 2016-02-04

**Authors:** Maria E. Francia, Jean-Francois Dubremetz, Naomi S. Morrissette

**Affiliations:** 1 Cell Cycle Regulation Laboratory, Instituto Gulbenkian de Ciencia, Oeiras, Portugal; 2 UMR 5235 CNRS, Université de Montpellier 2, Montpellier, France; 3 Department of Molecular Biology and Biochemistry, University of California Irvine, Irvine, CA USA

**Keywords:** Microtubule organizing center, Microgamete, Coccidia, Malaria, Centriole, Flagellum

## Abstract

The phylum Apicomplexa encompasses numerous important human and animal disease-causing parasites, including the *Plasmodium* species, and *Toxoplasma gondii*, causative agents of malaria and toxoplasmosis, respectively. Apicomplexans proliferate by asexual replication and can also undergo sexual recombination. Most life cycle stages of the parasite lack flagella; these structures only appear on male gametes. Although male gametes (microgametes) assemble a typical 9+2 axoneme, the structure of the templating basal body is poorly defined. Moreover, the relationship between asexual stage centrioles and microgamete basal bodies remains unclear. While asexual stages of *Plasmodium* lack defined centriole structures, the asexual stages of *Toxoplasma* and closely related coccidian apicomplexans contain centrioles that consist of nine singlet microtubules and a central tubule. There are relatively few ultra-structural images of *Toxoplasma* microgametes, which only develop in cat intestinal epithelium. Only a subset of these include sections through the basal body: to date, none have unambiguously captured organization of the basal body structure. Moreover, it is unclear whether this basal body is derived from pre-existing asexual stage centrioles or is synthesized de novo. Basal bodies in *Plasmodium* microgametes are thought to be synthesized de novo, and their assembly remains ill-defined. Apicomplexan genomes harbor genes encoding δ- and ε-tubulin homologs, potentially enabling these parasites to assemble a typical triplet basal body structure. Moreover, the UNIMOD components (SAS6, SAS4/CPAP, and BLD10/CEP135) are conserved in these organisms. However, other widely conserved basal body and flagellar biogenesis elements are missing from apicomplexan genomes. These differences may indicate variations in flagellar biogenesis pathways and in basal body arrangement within the phylum. As apicomplexan basal bodies are distinct from their metazoan counterparts, it may be possible to selectively target parasite structures in order to inhibit microgamete motility which drives generation of genetic diversity in *Toxoplasma* and transmission for *Plasmodium*.

## Basic phylogeny and apicomplexan life styles

Apicomplexans are unicellular protozoa that belong to kingdom Chromalveolata and the infrakingdom alveolata. Chromalveolates descended from a heterotrophic bikont (a bi-flagellated eukaryote), in which a secondary endosymbiotic event gave rise to a plastid-like organelle [1]. Members of the alveolata are characterized by cortical alveoli (flattened vesicles located between the plasma membrane and a network of subpellicular microtubules), and micropore [2]. They are further divided into phyla including ciliates, dinoflagellates, and apicomplexans, which differ mainly in their motile machinery [3, 4]. While ciliates and dinoflagellates move by means of cilia or flagella, invasive stage apicomplexans typically move by gliding motility. Additionally, many ciliates and dinoflagellates are free-living, while all apicomplexans are obligate parasites.

**Fig. 1:**
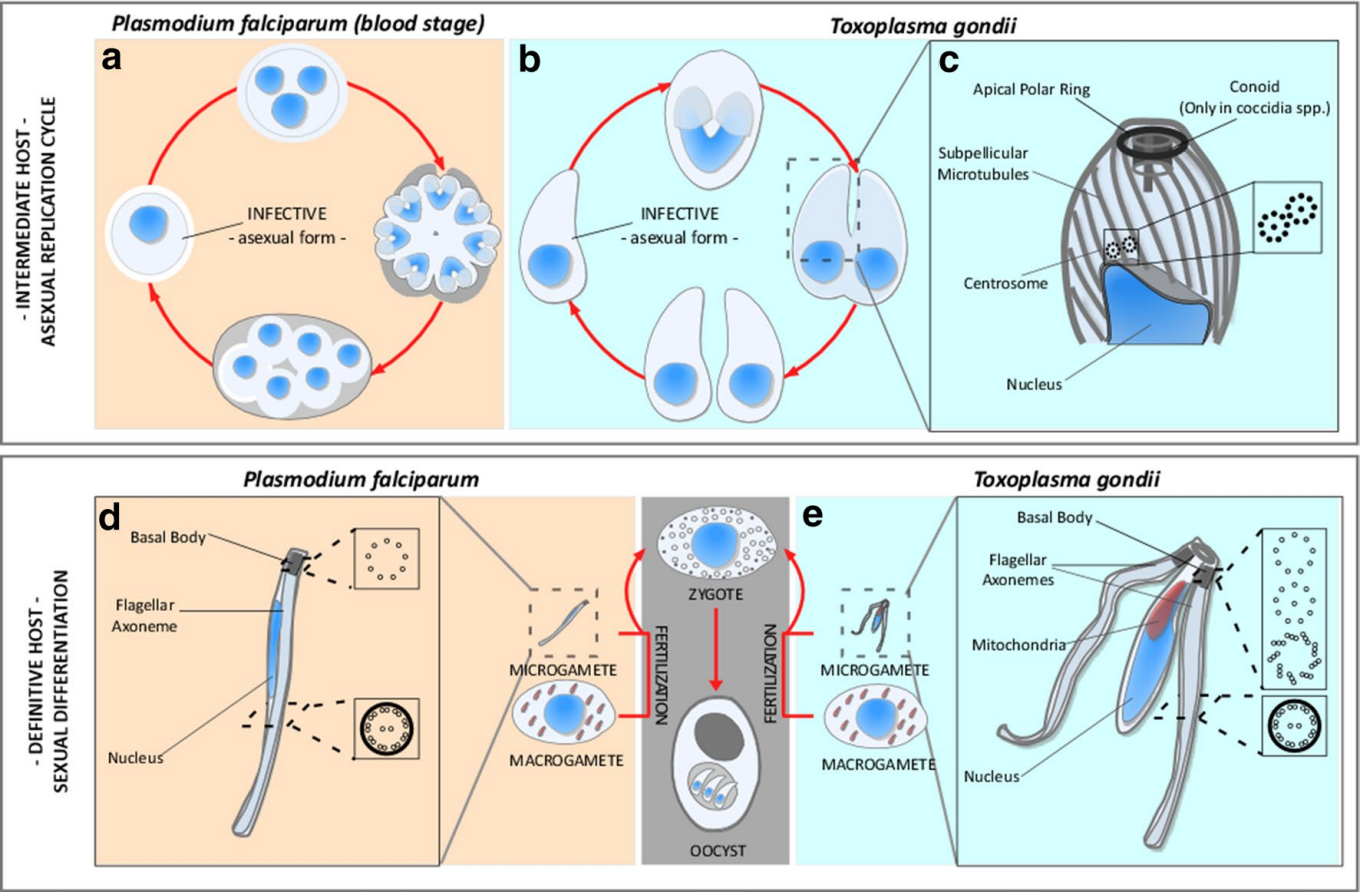
Life cycle and microtubule-based structures of apicomplexa. **a**–**e** Simplified schematic of the life cycle of Apicomplexa in their different hosts. Apicomplexa replicate either sexually or asexually. Differentiation into gametes and sexual replication occur within definitive hosts. Definitive hosts vary among apicomplexan species; *T. gondii* replicates sexually within felines, while *Plasmodium* species do so in mosquitoes. Flagellated forms of Apicomplexa are only found in definitive hosts, where they differentiate into male (micro) and female (macro) gametes. Fusion of gametes gives rise to a zygote which further differentiates into oocysts able to sporulate. Microgametes of different Apicomplexa vary in their number of flagella. *T. gondii* microgametes, represented here, have two protruding flagella. *Plasmodium* spp. microgametes emerge with a single flagellum upon terminal differentiation, and are assembled entirely within the cytoplasm of the undifferentiated originating cell. **a**, **b** In intermediate hosts, such as humans, apicomplexans grow vegetatively. Distinct replication modes among Apicomplexa allow them to adapt to different host niches. However, they all generate new infective zoites by assembly of daughter cells within the mother cell´s cytosol or at the mother cell surface, and undergo closed mitosis of the nuclear content. **c** Infective forms of Apicomplexa organize microtubules using functionally and physically distinct MTOCs. subpellicular microtubules, which impart shape and polarity to the cells, are organized by an MTOC localized at the apex, known as the APR. In addition, coccidian species in the phylum contain a specialized tubulin-based structure known as the conoid which has been evolutionarily linked to basal bodies of related flagellated alveolates [51, 52, 56]. Nuclear division occurs by closed mitosis. Chromosomes are organized by an intra-nuclear spindle nucleated by a cytosolic centrosome. Apicomplexa centriole-based centrosomes contain two centrioles of 9+1 singlet microtubule structure, oriented parallel to each other. Malaria-causing parasites (*Plasmodium* spp.) do not have canonical centrosomes, and organize their mitotic spindle from a “centriolar plaque” which can be identified using anti-centrin antibodies. The centriolar plaque is embedded in the nuclear envelope (not shown). **d**, **e** Microgamete flagella and basal body structures. Apicomplexa flagellar axonemes are composed of 9 doublet microtubules and a central pair [15–18]. **d** Basal bodies in malaria are better characterized, and consist of nine single A-tubules with no central tube, embedded in an electron-dense mass [16]. **e** Basal body structures are not well characterized in *T. gondii*. A small number of ultra-structural studies have led researchers to propose multiple alternative microtubule arrangements; a nine singlet microtubules, and a central tubule [20], atypical 9+0 and 9+2 arrangements, or a typical triplet microtubule structure with ninefold symmetry [8, 20, 22–26]

The phylum Apicomplexa encompasses numerous important disease-causing pathogens including the agents of malaria, toxoplasmosis, cryptosporidiosis, Texas and East Coast fever, and coccidiosis. The most widely studied of these are *Plasmodium* species, agents of malaria [5] and *Toxoplasma gondii*, which causes toxoplasmosis [6]. Apicomplexans undergo asexual (vegetative) replication in order to cause acute infection (Fig. 1a, b). During asexual replication, Apicomplexa use distinct cell division schemes of fascinating complexity and variability to adapt to different hosts and suit specific intracellular niches [7]. Both *Plasmodium* and *Toxoplasma* can also differentiate into gametes and undergo sexual recombination (Fig. 1d, e). In *Toxoplasma*, this occurs in the intestinal epithelium of cats and the resulting oocyst is shed in cat feces and sporulates in the environment. New infections are initiated by accidental ingestion of oocysts in contaminated food or water. *Toxoplasma* may also be transmitted by ingestion of infected animals harboring tissue cysts that contain latent asexual stage bradyzoite forms. *Plasmodium* gametes complete development and fuse to form a zygote in the stomach of mosquitos that have taken up infected blood. The zygote penetrates through the gut epithelium and undergoes meiosis and many rounds of replication to produce sporozoites. These migrate to the salivary glands and infect a new host when the mosquito takes another blood meal. Significantly, the sexual cycle is obligatory to natural transmission of most *Plasmodium* infections.

## Basal body organization in apicomplexans

Electron microscopy established early on that apicomplexan microtubule organizing centers (MTOCs) are structurally different from centrosomes found in most model systems. *Toxoplasma* has two juxtanuclear centrioles, arranged parallel to each other. These exhibit a 9+1 singlet microtubule symmetry, and are shorter than their animal counterparts (200 × 200 nm) (Fig. 1c) [7–11]. *Plasmodium* species appear to lack centrioles; instead, the spindle microtubules originate from a MTOC known as the “centriolar plaque” (CP) that is located within the nuclear envelope. The CP can be identified using antibodies to centrin [12–14]. The invasive asexual stages of apicomplexans are not flagellated, and therefore neither of these MTOCs functions as a basal body. In both *Toxoplasma* and *Plasmodium*, only the male gamete, known as the microgamete, assembles basal bodies and flagella (Fig. 1d, e).

**Fig. 2:**
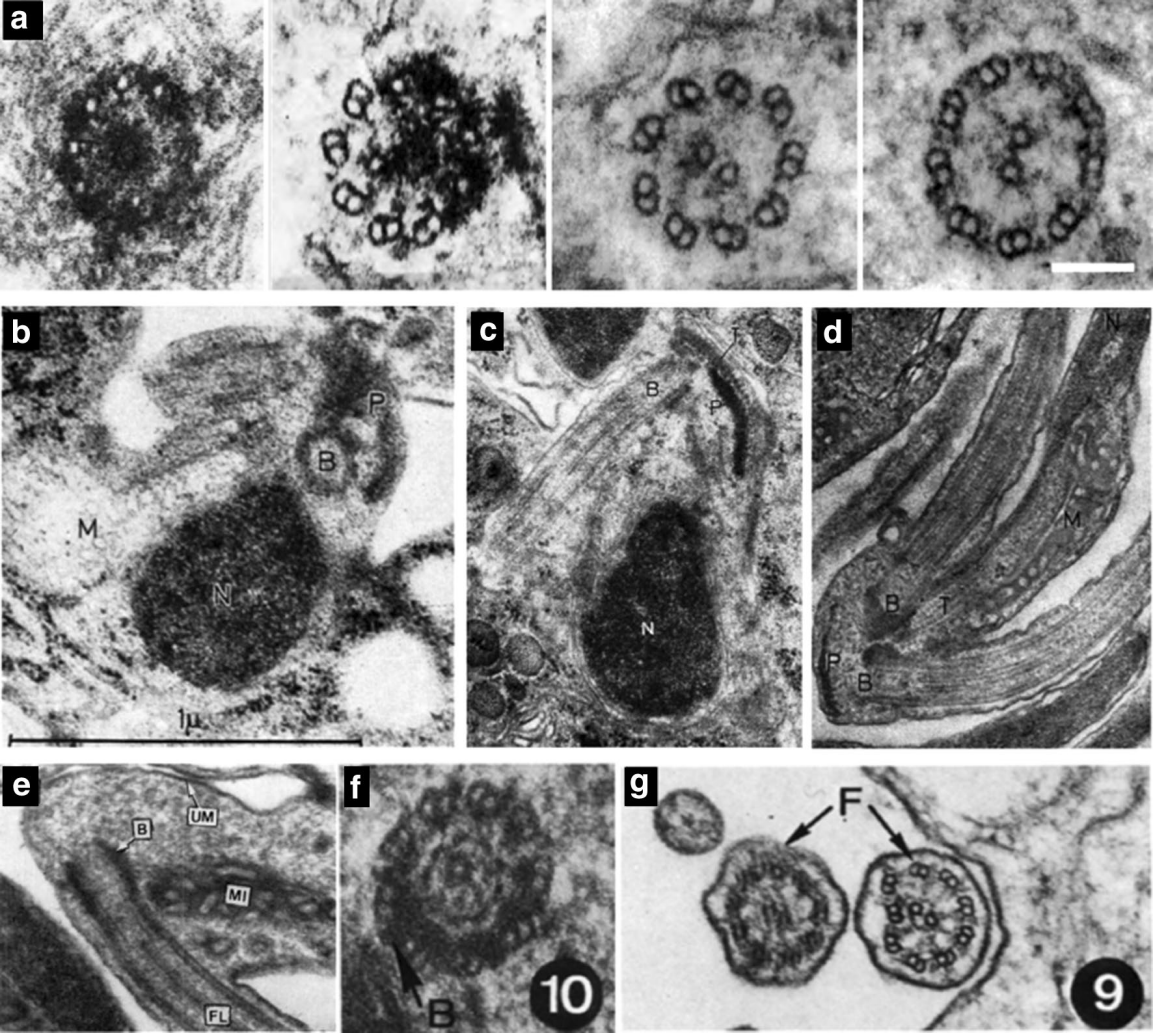
Basal Body and axoneme structures in *Plasmodium, Toxoplasma, and Sarcocystis*. **a** Serial transverse sections of the developing axoneme of a *P. falciparum* microgamete. From *left* to *right*: a basal body made of singlet microtubules, and embedded in an electron-dense mass and the distal flagellar region made of a 9+2 microtubule arrangement, can be observed. Reproduced from Fig. 1f in [16] with permission. **b** Transverse section through the pole (P) of a developing *T. gondii* microgamete. Several organelles are visible including a basal body (B) and the nucleus (N). **c** Longitudinal section through a *T. gondii* microgamete where one flagellum and its originating basal body (B) are visible. **d** Longitudinal section through a *T. gondii* microgamete where two flagella, and their originating basal bodies (B), are visible. **b**–**d** Reproduced from Fig. 2a, b, and d in [17], respectively. **e** Longitudinal section through the anterior portion of a *T. gondii* microgamete showing the position of the basal body (B) and flagella (FL) enclosed by a unit membrane (UM). Reproduced from Fig. 25 in [21]. **f** Transverse section through a basal body of a *Sarcocystis suihomin*is microgamete, a coccidian parasite closely related to *T. gondii,* showing some microtubules doublets and triplets (*arrow*, B). **g** Transverse section through two flagellar axonemes (*arrow*, F) of *S. suihomin*is. **f**, **g** Reproduced from Figs. 9 and 10 from [23], respectively

Mature *Plasmodium* sperm have a single flagella (Fig. 1d) while *Toxoplasma* microgametes are bi-flagellated (Fig. 1e). In both cases, the flagellar axoneme consists of 9 doublet microtubules and a central pair (Fig. 1d, e) [15–18]. Basal bodies in malaria consist of nine single A-tubules with no central tube, embedded in an electron-dense mass (Figs. 1d, 2a) (see Ref. [19] and Fig. 1f in Ref. [16]). The basal body microtubules extend 250 nm [19]. Basal body structure, however, remains somewhat unclear in *Toxoplasma*. *T. gondii* differentiates into gametes and reproduces sexually within felines. Therefore, microgamete isolation requires heavy infection of felid enteric tissue. This is technically challenging, and a limiting aspect to the study of this stage of the parasite life cycle. Moreover, transverse sections through the basal body barrel are uncommon in ultra-structural studies. The small number of images obtained to date suggests nine short singlet microtubules, and a central tubule basal body structure, which closely resemble the asexual form’s centriole structure (Fig. 2b–e) [17, 20, 21] (see Fig. 2a–d in Ref. [17], and Fig. 25 in Ref. [21]). On the other hand, studies of gametogenesis in closely related apicomplexans (other coccidian such as *Eimeria* and *Sarcocystis*) suggest that *T. gondii* basal bodies could consist of either typical triplet microtubule structure with ninefold symmetry (see Fig. 3b in Ref. [22] and Fig. 10 in Ref. [23] in *Eimeria* and *Sarcocystis*, respectively) or atypical 9+0 or 9+2 arrangements of singlet microtubules [8, 20, 22–26] (Figs. 1e, 2f). It is possible, however, that the latter correspond to serial sections through the transition zone or flagellar axoneme that are incorrectly interpreted as basal bodies.

The *Toxoplasma* genome (available at toxodb.org [27]) contains genes for three α- and β-tubulin isotypes [28, 29]. Inferences from proteomes and mRNA data suggest that all isotypes are expressed at some levels in asexual forms and in oocysts [30] (Fig. 1a). The *T. gondii* genome also contains a single γ-tubulin gene, which has been shown to localize to centrioles [30, 31]. Both δ- and ε-tubulin isoforms appear to be present in the genome; however, publically available mass spectroscopy data (toxodb.org) do not provide evidence for expression in asexual forms. Therefore, *T. gondii* is likely equipped with the necessary elements to assemble triplet microtubule blades of basal bodies [32–36]. Whether δ- and ε-tubulin genes are expressed specifically in microgametes remains an intriguing question, as this could explain the developmental maturation of singlet centrioles into triplet basal bodies that template flagellar axonemes [30]. Unfortunately, to date, expression data are not available for *T. gondii* gametes. In addition to α-, β-, and γ-tubulin genes, *P. falciparum* has single apparent homologs of δ- and ε-tubulin (PF3D7_1475700 and PF3D7_0933800, respectively), and neither is expressed at significantly higher levels during gametogenesis (expression data available at plasmodb.org).

## Microtubule organizing centers during the parasite life cycle

Apicomplexans use spatially and morphologically distinct microtubule organizing centers (MTOCs) to functionally organize independent microtubule subsets. Cell shape and polarity is organized by subpellicular microtubules nucleated by a ring-shaped MTOC localized at the cell apex, known as the apical polar ring (APR) (Fig. 1c). The APR consists of concentrically arranged tubulin rings, and a central pair of microtubules [37]. Microtubules organized by the APR emerge in comma-shaped fashion and extend two-thirds into the cell length. Coccidian apicomplexans, including *T. gondii*, also construct an additional tubulin-based structure known as the conoid [8]. The conoid is composed of fourteen tightly apposed tubulin-based filaments that spiral counterclockwise toward the pre-conoidal rings [8, 38]. It can be extended from or retracted into the APR. Though no direct evidence for its function has been demonstrated, a role in host cell invasion has been frequently ascribed to this structure [37]. *Plasmodium* merozoites have a dramatically reduced set of subpellicular microtubules, also organized from an APR [39]. Nuclear division occurs by closed mitosis: the nuclear envelope remains intact and spindle microtubules are inserted into pores in the nuclear envelope. In coccidians such as *T. gondii*, spindle microtubules are organized by a specialized structure known as the centrocone. The centrocone is a protrusion in the nuclear envelope associated with the cytoplasmic centrioles (Fig. 1c).

## Basal body origins

In *T. gondii*, basal bodies have been proposed to either form de novo or to be derived from the pre-existing centrioles [18]. However, because the basal body structure remains undefined, a number of hypothetical biogenesis pathways can be proposed. If basal bodies are composed of nine singlet microtubules and a central tube, pre-existing centrioles could become basal bodies directly. It is also possible that the singlet microtubule-based centrioles mature into triplet microtubule-based basal bodies by gamete-specific expression of δ- and ε-tubulin genes, giving rise to a more typical basal body configuration. Additionally, basal bodies could be synthesized de novo as is proposed to be the case in *Plasmodium*, in which case the alternative 9+0 and 9+2 singlet structures reported could be generated.

*Plasmodium* sexual differentiation occurs in the midgut of the female mosquito vector [16]. The signals that trigger differentiation and flagella formation (exflagellation) can be mimicked in vitro. Time course studies of the exflagellation process suggest that the basal body in *Plasmodium* forms de novo, but its position is defined by the pre-existing CP. Molecular data on how this process is regulated are lacking, but ultra-structural TEM studies suggest that the centriolar plaque and the emerging basal body are functionally and physically linked. A single study in the malaria-related parasite *Haemoproteus columbae* shows that the electron-dense mass of the centriolar plaque houses a single centriole made of singlet microtubules and a central pair prior to the appearance of flagella [16, 40]. This has been proposed to be an “intermediate” step in the formation of the basal body, which physically separates from the CP at a later stage, but retains a 9+1 singlet microtubule architecture [16]. Consistent with the suggestion that *Plasmodium* basal bodies are formed de novo during microgametogenesis, expression of the conserved centriole component SAS-6 is restricted to microgametes [41]. Moreover, SAS-6 knock-out parasites do not form motile microgametes [41].

Additional basal body structures or accessory structures have not been described in the literature thus far. Gene expression data or proteomes from distinct stages of gametogenesis are not available, therefore limiting our understanding of the basal body and flagellar structural components and biogenesis pathways. Clear homologs of key regulators of centriole biogenesis in other eukaryotes, such as PLK4 or PLK1, are missing from the apicomplexan genomes [42, 43]. Therefore, the biogenesis of centrioles and basal bodies is expected to be distinct from other conserved assembly pathways. In fact, recent studies described divergent regulatory elements participate in the duplication of the *T. gondii’s* centrosome, suggesting that the centrosome replication pathway in Apicomplexa is non-canonical. A MAP kinase homolog (TgMAPK-L1, [31]) and a NIMA-related kinase (TgNEK1–2, [44]) were shown to play critical roles in duplication and maintenance of the “right number” of centrosomes structures in *T. gondii*. However, the exact mechanism by which they participate in centrosome duplication remains to be deciphered. In addition, an aurora kinase homolog (TgARK1) associates with centrosomes specifically in S-phase in *T. gondii*, when centrosome duplication occurs, suggesting that it too could play a role in the process [31]. *T. gondii* centrosomes are organized into two independent functional segments, named “cores.” These cores are distinguishable in composition, and each of them coordinates functionally distinct aspects of cell division allowing the parasite for cell cycle flexibility and adaptability. The “inner core” facing the nuclear envelope coordinates chromosome segregation. Meanwhile, the “outer core” localizes distal to the nucleus, and coordinates the assembly of new daughter cells [31].

## Identification of basal body components

Microgamete-specific proteomic studies have been achieved in *Plasmodium* because this stage is experimentally accessible, unlike the situation in *Toxoplasma* [45, 46]. These studies focused on identifying flagellar axoneme components, and while the results are informative for making inferences about axoneme properties, information on basal body components remains scarce. *Plasmodium* species lack intraflagellar transport (IFT) machinery [16, 47], and are devoid of most BBsome components which coordinate signaling functions in other flagella and cilia [48]. The *T. gondii* genome contains homologs of CEP164, BBS5, and IFT components, suggesting that IFT drives axoneme assembly [48]. These differences suggest that interesting differences in flagellar assembly pathways between these apicomplexans could exist: *Plasmodium* assembles flagella within the cytosol, while *T. gondii* microgamete´s flagella are likely to be extruded from the periphery.

Both *T. gondii* and *Plasmodium* genomes lack many basal body components. However, they encode the “UNIMOD” components SAS6, SAS4/CPAP, and BLD10/CEP135 [16]. Additionally, homologs of Meckelin (MKS3), a conserved protein linked to Meckel Syndrome, is present in the genomes of apicomplexans [48]. As MKS3 assists centriole migration to the cell surface prior to flagella formation, this supports the model that pre-existing centrioles directly act as basal bodies. *T. gondii*, but not *Plasmodium*, encodes a VFL1 homolog [48], which establishes basal body orientation in the unicellular green alga Chlamydomonas [49]. Nonetheless, whether these components are expressed or indeed participate in basal body and flagellar assembly is unknown, as the roles of these apparent homologs have not been studied in apicomplexans. It is possible that these genes represent remnants of an ancestral flagellar assembly pathway that has been discarded by *T. gondii*. Molecular data supporting this alternative hypothesis are discussed below.

## Notable basal body findings

The infectious asexual forms of Apicomplexa do not assemble flagella and therefore lack basal bodies. Nevertheless, the microtubule-based structures present in apicomplexan asexual forms pose interesting evolutionary questions. *Plasmodium* and *Toxoplasma* genomes encode both a canonical SAS-6, which localizes to the centrosome [30], and a smaller protein of conserved N-terminal called SAS6-like (SAS6L). Canonical SAS6 self-assembles in vitro into geometrically defined structures, and this is thought to template the formation of the centriole cartwheel [50]. Overexpressed SAS6L assembles into filaments in a microtubule-independent fashion [51]. In *Toxoplasma* tachyzoites, SAS6L localizes to the conoid at the parasite apex, some distance from the juxtanuclear centrioles [51]. Interestingly, the *Plasmodium* SAS6L homolog is up-regulated in gametocyte development suggesting that it may play a role in basal body assembly in non-coccidian apicomplexans. The conoid also establishes a physical connection with proteins known as striated fiber assemblins (SFAs) [52]. In flagellates, SFAs contribute to the basal body rootlet system which orients the basal bodies relative to other subcellular structures [52, 53]. Apicomplexan SFAs are highly similar to SFA in flagellated algae such as Chlamydomonas [52, 54]. However, apicomplexan SFAs are expressed in the absence of flagella during asexual replication. Apicomplexan cell division occurs by internal assembly of daughter cells within the cytosol or at the surface of the mother cell [7]. These SFA fibers form immediately after centriole duplication and establish a physical link between the duplicated centrioles and the emerging daughter APR and conoid structures [52]. This connection is essential to ensure the proper segregation of genetic material to the emerging daughter cells, as the centrioles remain durably connected to the chromosomes by way of the mitotic spindle throughout cell division [7, 55]. Interestingly, the non-apicomplexan alveolates *Colpodella vorax* and *Rastrimonas subtilis* construct pseudoconoids which are somewhat similar to conoids. Unlike in apicomplexans, these organisms build flagella adjacent to an apical pseudoconoid structure. It has been proposed that in adapting to parasitic lifestyles apicomplexans lost their flagella. However, the basal body and the conoid may be linked by historical interdependence. We and others have hypothesized that the non-flagellated forms of apicomplexans may have maintained ancestral basal body accessory structures to aid in organelle inheritance during cell division [51, 52, 56].

## Strengths and future of basal body research in Apicomplexa

Basal bodies with associated flagellar axonemes are assembled only during microgamete formation in the sexual stage of the parasite life cycle. The sexual stages of apicomplexans are of great interest to the research community as they are required for generating genetic variation in both *T. gondii* and *Plasmodium*. They are also required for transmission of malaria in endemic areas. To date, we know that basal bodies are structurally and compositionally different from their mammalian counterparts. It is likely that their precise composition, organization, and construction are all distinct. However, our molecular insight into these structures remains superficial. Tubulin-based structures have been validated as anti-parasitic targets in *T. gondii* [30], and blocking transmission in mosquitoes has been proposed as a viable route for malaria prevention [16, 41]. Better understanding of the molecular components and biogenesis of apicomplexan basal bodies and flagellar axonemes is critical to meeting these goals. Obtaining microgametes in vitro is technically challenging, and currently represents the major rate limiting step in the advance of our molecular understanding of these cells. Efficient technology to promote the differentiation of asexual forms into microgametes in vitro or ex vivo could greatly impact our ability to understand differentiation at the structural and molecular level of these important human pathogens. Efforts focusing in developing these technologies should be at the center of Apicomplexa basal body research in the future.

## Abbreviations

MTOC: microtubule organizing center
CP: centriolar plaque
BBS: Bardet–Biedl syndrome
IFT: intraflagellar transport
EM: electron microscopy
APR: apical polar rings
SFA: striated fiber assembly
SAS6-L: SAS6-like
